# The Landscape of Adoptive Cellular Therapies in Ovarian Cancer

**DOI:** 10.3390/cancers15194814

**Published:** 2023-09-30

**Authors:** Lucy Davis, Rowan E Miller, Yien Ning Sophia Wong

**Affiliations:** 1 Royal Free Hospital, London NW3 2QG, UK; lucy.davis22@nhs.net; 2 Department of Medical Oncology, University College London Hospital, London NW1 3PG, UK; rowan.miller@ucl.ac.uk; 3 Department of Medical Oncology, St Bartholomew’s Hospital, London EC1A 7BE, UK

**Keywords:** adoptive cellular therapy, ovarian cancer, immunotherapy, CAR-T therapy, TCR-T therapy, TIL, cancer vaccine

## Abstract

**Simple Summary:**

Adoptive cellular therapy (ACT) is a subset of immunotherapy that offers a compelling personalised alternative to immune checkpoint inhibition. However, there are challenges to be overcome in the use of ACT in solid organ malignancies and their investigation is currently in the early stages. In this review, we aim to discuss the benefits and challenges of available modalities of ACT in ovarian cancer, presenting the results of clinical trials and the rationale for upcoming studies.

**Abstract:**

Ovarian cancers are typically poorly immunogenic and have demonstrated disappointing responses to immune checkpoint inhibitor (ICI) therapy. Adoptive cellular therapy (ACT) offers an alternative method of harnessing the immune system that has shown promise, especially with the success of chimeric antigen receptor T-cell (CAR-T) therapy in haematologic malignancies. So far, ACT has led to modest results in the treatment of solid organ malignancies. This review explores the possibility of ACT as an effective alternative or additional treatment to current standards of care in ovarian cancer. We will highlight the potential of ACTs, such as CAR-T, T-cell receptor therapy (TCR-T), tumour-infiltrating lymphocytes (TILs) and cell-based vaccines, whilst also discussing their challenges. We will present clinical studies for these approaches in the treatment of immunologically ‘cold’ ovarian cancer and consider the rationale for future research.

## 1. Introduction

The discovery of immune checkpoint inhibitors (ICIs) has been transformative in the treatment landscape of several solid cancers [1]. However, responses to ICI therapy have been modest in ovarian cancer (OC), with some phase III trials prematurely terminated due to futility [2,3,4,5,6,7,8]. The development and use of cell-based adoptive immunotherapies has accelerated in recent years [9] due to a variety of factors, including substantial insights into and understanding of the immune landscape of the tumour microenvironment (TME), novel techniques to identify useful host immune cells such as neoantigen reactive T-cells, and the technology to manufacture a cellular product for safe and reproducible delivery to patient populations.

### Adoptive Cellular Therapy in the Era of Personalised Medicine

The early pioneering work of Steve Rosenberg’s team in extracting and expanding anti-cancer immune cells, specifically T-lymphocytes in the 1980s, has laid the foundations for the use of adoptive cell transfer in cancer immunotherapy [10]. Broadly, adoptive T-cell therapy has two main approaches—using tumour-infiltrating lymphocytes (TILs) or lymphocytes modified to target tumour-associated or tumour-specific antigens [11].

**Figure 1:**
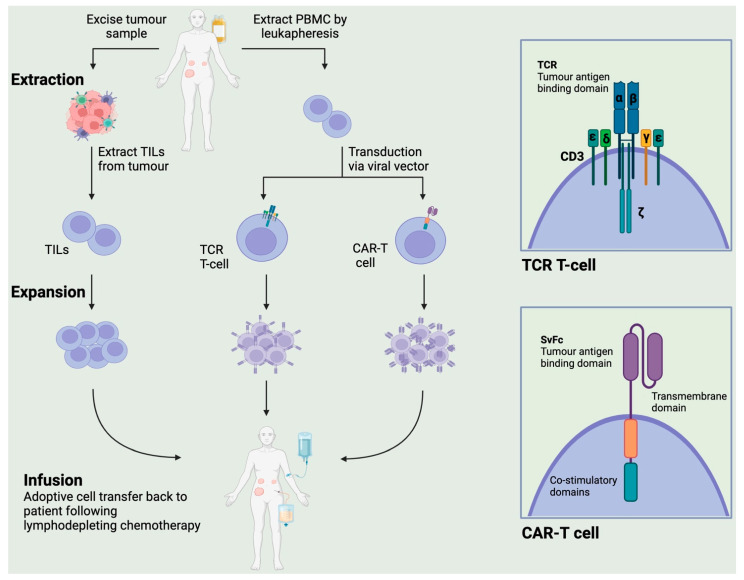
Schema of adoptive cellular therapies. Tumour-infiltrating lymphocytes (TILs) from the patient’s tumour sample can be harvested, expanded *ex-vivo* and reinfused back to the patient. T-lymphocytes can be extracted as peripheral blood mononuclear cells (PBMCs) by leukapheresis and genetically modified to express a T-cell receptor (TCR) or a chimeric antigen receptor (CAR), this cell population is then expanded and reinfused back to the patient. TCR-T cells express the T-cell receptor specific to tumour-associated antigens along with associated costimulatory domains. CAR-T cells express a chimeric antigen receptor with an svFc region specific to tumour-associated antigens and intracellular costimulatory domains originating from the TCR structure.

Lymphocytes can be extracted from tumours, expanded *ex vivo* and then reinfused into patients as TIL therapy, often in conjunction with lymphodepleting (LD) preconditioning regimens (Figure 1). In many situations, an approach using TILs is not possible, either due to a lack of TILs within the tumour sample or insufficient biopsy material. Alternatively, peripheral lymphocytes can be engineered to enhance their specificity to the patient’s tumour, somewhat overcoming the limitation of paucity of infiltrating lymphocytes within the tumour itself. Peripheral blood lymphocytes are typically harvested before undergoing genetic modification to express receptors that confer specificity to a tumour antigen.

Adoptive cell therapy (ACT) using engineered lymphocytes involves either the introduction of genes for the antibody-based chimeric antigen receptor (CAR) or the traditional T-cell receptor (TCR), although this strategy is restricted to antigens presented by the major histocompatibility complex (MHC) [12] (Figure 1). One major advantage of CARs over TCRs is their ability to target tumour antigens in an MHC-unrestricted manner. Furthermore, CARs have the ability to bind to a range of cell surface such as glycosylation variants rather than being limited to protein antigens, which may widen the potential for tumour-specific antigenic targets [13].

In this review of ACT, we will discuss the role of emerging cell-based immunotherapy strategies in solid organ malignancies using OC as an exemplar. We will detail the biological insights behind recent developments for ACT in OC and present the results of clinical studies as well as possible future directions.

## 2. Types of Adoptive Cellular Therapy (ACT)

### 2.1. Chimeric Antigen Receptor T (CAR-T) Therapy

A chimeric antigen receptor T (CAR-T) cell consists of a T-lymphocyte that has been modified *in vitro* to express a specific antigen receptor. A CAR is itself a fusion of a single-chain variable region capable of recognising tumour-associated antigens and intracellular portions of the T-cell receptor that have the crucial property of transducing the signal to activate T-lymphocytes [14]. There is a wealth of evidence that CAR-T cells have been used successfully to improve outcomes for patients with haematologic malignancies [15,16,17]. Their unique properties are now being exploited in the treatment of solid organ cancers including ovarian cancer.

Challenges of CAR-T cells in OC include the heterogenous nature of these tumours, leading to difficulty identifying antigens that are tumour-specific yet sufficiently expressed to induce a response. Lack of persistence and trafficking of CAR-T cells *in vivo* has been an additional barrier to maximising clinical response rates [18]. This is likely due to the hostile TME, poor vascular integrity, and downregulation of adhesion molecules [19]. It is known that immunosuppressive mechanisms are at play in the TME, including the presence of regulatory T-cells and cytokines that may attenuate the effect of CAR-T cells.

In terms of side effects, there are hurdles to be overcome in the use of CAR-T cells in solid organ malignancies. Target antigens are often expressed to varying degrees in healthy tissues and the phenomenon of the ‘on-target, off-tumour’ effect is observed when CAR-T cells destroy healthy tissue and give rise to adverse clinical effects [20]. A further potential toxicity of this treatment is the cytokine release syndrome (CRS). CRS presents with a spectrum of severity ranging from flu-like symptoms to circulatory shock due to the massive release of cytokines that can occur on lymphocyte activation. Earlier clinical trials in this area focused on the safety profile of CAR-T therapy and how this is affected by co-administration of other systemic anticancer agents.

A variety of tumour-associated antigens have been identified in OC as suitable targets for CAR-T cells and formed the basis of phase I and II clinical trials in recent years. A popular antigenic target utilised in CAR-T cell therapy in OC is mesothelin, a membrane glycoprotein expressed in particularly high levels in serous epithelial ovarian cancers as well as normal mesothelial tissues [21]. It has been reported that non-specific toxicity rates are lower due to low-level expression of mesothelin in normal tissues, making it an attractive target for CAR-T therapy [22]. In 2014, Haas et al. showed that mesothelin-directed CAR-T cells can persist in the peripheral blood, with peak levels at 6–14 days following a single infusion in 19 subjects with mesothelin positive tumours, including five with OC [23]. This expansion *in vivo* was enhanced with the administration of cyclophosphamide, suggesting that LD chemotherapy prior to administration of CAR-T cells may serve to potentiate their anti-cancer effect. Wang et al. reported similar peak serum levels of CAR-T directed against mesothelin at 7–14 days in 15 subjects, including one OC patient with mesothelin-positive tumour tissue [24]. In contrast to the Haas et al. [23] study, the single CAR-T cell infusion was given without any LD chemotherapy. Additionally, none of these subjects reported adverse events of any grade (Table 1). When administered with LD chemotherapy and IL-2, mesothelin CAR-T cells have been associated with progressive disease (PD) in 14/15 patients with mesothelin-positive tumours at 3.5 months (NCT01583686; Table 1). Multiple trials are still ongoing to assess the safety and efficacy of mesothelin-directed CAR-T cells in patients with advanced OC (Table 2).

A well-known biomarker of OC is CA-125, which is itself a domain of a large transmembrane glycosylated protein called MUC16 that has become a focus of CAR-T therapy in OC over recent years [35]. MUC16 has been shown to be overexpressed in ovarian cancers and linked to promoting cell proliferation, migration and invasion [36]. O’Cearbhaill et al. published a study in 2020 in which 18 heavily pretreated patients with ovarian, fallopian tube and primary peritoneal cancers received CAR-T cells directed at MUC16 and engineered to secrete IL-2 [25] (Table 1). They were able to first establish a maximum tolerated dose that was then administered both intravenously and intraperitoneally to a cohort of patients after receiving LD chemotherapy. A best response of stable disease (SD) was observed, along with serum levels of CAR-T cells peaking at 7–28 days. However, two of the three patients who received LD chemotherapy experienced dose-limiting toxicity (DLT) in the form of macrophage activation-like syndrome, leading to this cohort being closed for further recruitment. Although the sample size was small, these data suggest that LD chemotherapy may increase the risk of toxicity, especially in the context of IL-2 secretion. Looking forward, an ongoing study (NCT03907527) is recruiting a similar cohort of patients with advanced, platinum-resistant OC at an estimated larger sample size of 71 subjects. They are planned to receive CAR-T cells directed at MUC16 either intravenously or intraperitoneally and with or without prior LD chemotherapy. This should allow for comparison of maximum tolerated dose and efficacy between these different routes of administration (Table 2).

An additional protein that has been studied as a target for CAR-T cells is the folate receptor alpha (FRα), which has been shown to be expressed in at least half of ovarian cancers of a diverse range of phenotypes [37] and of limited expression in healthy tissues [38]. A pioneering study treated subjects with recurrent epithelial OC with CAR-T cells directed at FRα. They were able to demonstrate safety when given alone but showed grade 3–4 adverse effects when co-administered with IL-2 [26] (Table 1). Furthermore, no objective reduction in tumour burden was seen in these patients, likely linked to the low levels of radio-labelled CAR-T cells detected in the tumour and peripheral blood after a few weeks. Currently ongoing is a study (NCT03585764) that is recruiting those with persistent and pretreated high grade serious ovarian cancer (HGSOC) to receive FRα CAR-T cells via the intraperitoneal route and with or without LD chemotherapy. Perhaps it will find that the addition of lymphoreductive treatment is associated with increased persistence of the FRα CAR-T cells and better anti-tumour response (Table 2). The transmembrane glycoprotein B7-H3 is highly expressed in human OC and has shown a correlation with CD8 [39]. CAR-T cells directed at B7-H3 are currently being evaluated in cohorts of patients with epithelial OC (NCT04670068) and recurrent OC (NCT05211557). Other CAR-T targets, such as TAG-72 (NCT05225363) and ALPP (NCT04627740), are currently under investigation in clinical trials (Table 2).

### 2.2. Genetically Engineered T Cell Receptor (TCR) Therapy

TCR therapy consists of, in most cases, peripheral blood lymphocytes that have been modified to express the T-cell receptor specific to a certain cancer-associated antigen. In contrast to CAR-T cells, TCR-T cells recognise cancer antigens in an MHC-restricted manner and these targets tend to be of the cancer testis antigen (CTA) type, often consisting of normal developmental proteins whose expression has been abnormally upregulated in malignant tissue [40]. An advantage of TCR-T cells over CAR-T cells is their ability to also target intracellular antigens, which may increase their cytotoxic and anti-tumour activity.

A popular CTA target for TCR-T cells in solid tumours is the melanoma-associated antigen (MAGE), which has been shown to be overexpressed in a high percentage of OCs and in some reports has been associated with decreased progression-free survival [41]. A phase I study treating patients with a range of MAGE-A4-positive tumours following LD chemotherapy has reported results in 2023 [27] (Table 1). Grade 3–4 haematological toxicity was observed in all patients, leading to an adjustment of the LD chemotherapy dose. Two trial-related deaths were recorded, including one OC patient who experienced grade 3 neurological toxicity followed by death due to cerebrovascular accident. Overall, 74% of the patients had SD or partial response (PR), including five with OC. A sub-study of this trial is underway to explore whether the use of radiotherapy may work synergistically with TCR therapy with a more favourable adverse effects profile than LD chemotherapy. A phase II randomised study (NCT05601752; Table 2) is currently recruiting participants with recurrent OC to receive TCR-T cells directed against MAGE-A4 as a monotherapy or with the immune checkpoint inhibitor (ICI) Nivolumab. As well as providing the safety and efficacy data of this combinatorial approach, this study may also help to characterise surrogate markers of treatment effect in this heavily pretreated population.

A commonly studied CTA, which has been the focus of some early-phase TCR-T clinical trials in OC over recent years is the New York Esophageal-1 antigen (NY-ESO-1). This antigen has been found to be expressed in around 40% of a diverse range of OC tumours and higher expression has been associated with later stages, poorer response to initial treatment and a serous phenotype [42]. A 2016 study (NCT02869217) has recruited participants with a wide range of NY-ESO-1-positive solid tumours to receive cyclophosphamide followed by either a single dose or repeated intravenous doses of TCR-T cells directed at NY-ESO-1. Early results have so far shown mild CRS and a small amount of G3–G4 toxicity following a single TCR-T infusion. More promisingly, out of the nine participants treated by 2019, two had a PR and another five had SD [28] (Table 1).

The Roswell Park Cancer Institute has conducted multiple trials with NY-ESO-1-targeted TCR-T cells in patients with recurrent epithelial ovarian, primary peritoneal or fallopian tube carcinoma and platinum-refractory or -resistant disease. Six participants in an early trial beginning in 2012 received low dose LD chemotherapy followed by a single IV infusion of NY-ESO-1 TCR-T cells. No appreciable reduction in tumour burden was seen and, furthermore, five of the six patients reported serious adverse effects [29] (Table 1). A number of techniques have been employed in later studies to try and enhance the anti-tumour effect of NY-ESO-1 TCR-T cells. For example, one trial (NCT03017131) utilised the pyrimidine nucleoside analogue Decitabine as well as IL-2 with TCR-T cells, aiming to measure toxicity, persistence of TCR-T cells and disease response. In addition to this, researchers plan to sample tumours in any disease recurrence following TCR-T treatment to assess for MHC/NY-ESO-1 levels and better understand treatment resistance mechanisms. An ongoing third study by the same group (NCT03691376) employs a Melphalan myeloablative conditioning regimen before intravenous autologous TCR lymphocytes and haemopoietic stem cells directed against NY-ESO-1 (Table 2) to assess whether this strategy increases TCR survival and clinical response.

A novel approach using TCR-T cells in solid cancers, including OC, is being trialled with an estimated enrolment of 271 participants in a phase II study that started in 2018 (NCT03412877). The most tumour-reactive TILs from each participant’s tumour sample will be selected, their TCR isolated and genetically expressed in autologous peripheral blood lymphocytes to create a TCR-T product. This personalised approach aims to combine the specificity of TIL with the cytotoxicity of effector T cells. One cohort will also receive PD-1 blockade with Pembrolizumab to assess whether this strategy can further enhance survival of TCR-T cells and improve response rates.

### 2.3. Tumour-Infiltrating Lymphocytes

Tumour-infiltrating lymphocytes have become increasingly recognised over recent years as playing an important role in the growth and control of solid tumours. TIL therapy, unlike engineered T-cell therapy, is associated with fewer incidences of CRS or immune effector cell-associated neurotoxicity (ICANS), resulting in a more favourable toxicity profile [43]. The use of high-dose non-myeloablative lymphodepletion (NMA-LD) and IL-2 post-transfer has, however, been linked to high-grade adverse events associated with TIL therapy.

NMA-LD is frequently reported to be associated with high-grade haematological adverse events, whilst recombinant IL-2 can lead to multi-organ toxicities, including heart, kidneys and lungs, as well as systemic toxicity, such as capillary leak syndrome [44]. Although these aspects of TIL therapy often lead to a requirement for inpatient management with specialist support, in most cases these high-grade toxicities occur acutely and can be addressed within the same inpatient admission [44,45]. Additionally, the ‘one-off’ nature of TIL therapy, in contrast to the majority of current standard-of-care anti-cancer approaches, makes it less time-consuming for patients and avoids the need for frequent appointments.

A 2017 prospective study by Goode et al. of over 5500 patients with OC found that TILs were often present in the epithelial tumour islets, with the highest infiltration found in HGSOC [46]. Importantly, an association was observed between increased levels of TILs and overall survival, which was most significant in the HGSOC group. These cells can be harvested from the patient’s tumour tissue, expanded *ex vivo* and transferred back to them as a form of ACT. A key difference between this approach and CAR-T cells and TCR-T cells is the reliance on pre-existing TILs in the patient’s tumour sample and the challenge of expanding this cell population *ex vivo*.

Earlier trials in the 1990s compared giving TILs vs. TILs with chemotherapy and showed some promising results. For example, Aoki et al. reported complete response in seven out of ten patients with advanced epithelial OC who received TILs with a cisplatin-based chemotherapy regimen [30] (Table 1). Following on from this, Fujita et al. conducted a study to compare TIL plus standard chemotherapy vs. standard chemotherapy alone in the adjuvant setting following primary debulking of epithelial OC [31] (Table 1). A significantly greater long-term survival rate was observed in the TIL group, suggesting that TILs could play a crucial role in preventing disease recurrence in epithelial OC and work synergistically with standard chemotherapy.

Much like endogenous T-cells, transferred TILs will be susceptible to the well-characterised immune regulation at play in the TME, which may be overcome by using combination therapy with ICIs [47]. The National Center for Cancer Immune Therapy has produced several clinical trials using TIL in OC patients, including in combination with ICIs. An initial pilot trial treated six platinum-resistant OC participants with TILs following LD chemotherapy and found 3–5 month SD in most, followed by progression [32] (Table 1). Of note, they observed high levels of TIL ‘exhaustion markers’ such as PD-1 and LAG-3 post infusion, as well as increased expression of MHC-II and PD-L1 in the tumour tissue, which may be impeding the efficacy of the treatment. Based on these results, a similar follow-up study of six patients was conducted with the addition of the ICI Ipilimumab prior to TIL harvest. At 12 months, five participants had SD and one had PR, indicating that the addition of ICIs may increase TIL yield and augment the cytotoxic effect of these cells in the tumour (Table 1). Analysis of the tumour samples again found expression of the inhibitory coreceptor LAG-3, which is known to interact with MHC-II on tumour cells. This was thought to be an additional target that could be utilised in improving the outcome of TIL therapy. A third study began in 2021 (NCT04611126) with an estimated recruitment of around 18 participants with advanced ovarian, fallopian tube and primary peritoneal cancer (Table 2). One cohort will receive the same regimen of ICI along with TILs whilst another cohort will also receive the LAG-3 inhibitor Relatlimab with PD-1 inhibitor Nivolumab. This trial remains in the recruitment phase and aims to provide data on safety profile and response rates of this combinatorial approach.

A similar approach is being adopted in an ongoing study (NCT03158935; Table 2) in which a cohort of patients with platinum-resistant OC will receive TILs followed by the PD-1 inhibitor Pembrolizumab [34]. An additional trial with an estimated enrolment of 15 participants with epithelial OC, fallopian tube or peritoneal cancer is currently recruiting (NCT03412526; Table 2). Participants will receive LD chemotherapy followed by a single treatment of 2Gy whole-body radiotherapy before infusion of TILs and recombinant IL-2. It has been shown in other tumour types that radiotherapy may increase the number of CD3^+^ and CD8^+^ TILs present in the tumour [48], so this study may provide an additional method of increasing TIL yield in OC. The use of interferon alpha is also being evaluated as a method of increasing TIL persistence and efficacy in an ongoing clinical trial (NCT04072263; Table 2).

## 3. Vaccines (Cell-Based)

Although not an ACT as such, vaccines can provide an alternative method of harnessing the host cellular and humoral immune system to target cancer-associated antigens. Clinical trials over the last 20 years have tested vaccines against OC antigens in both the adjuvant setting and in those with recurrent or persistent disease. Advantages to vaccine therapy include vaccines’ potential to induce immunity against multiple antigens, which may be particularly beneficial in OC owing to their heterogeneity. Vaccines may also be useful to enhance the effects of other immunotherapy agents, such as ICIs.

Vaccines have been studied in the setting of advanced metastatic OC as a method of achieving disease control, often used in conjunction with other modalities of treatment. A novel approach using dendritic cell-based vaccine in combination with a VEGF inhibitor, Bevacizumab, with or without cyclophosphamide, was adopted by Tanyi et al. in a trial that began in 2010 involving 25 platinum pre-treated participants with recurrent advanced epithelial OC [49]. They reported a median time to disease progression of 15 months amongst those patients who had objective T-cell vaccine responses. Furthermore, the group that received cyclophosphamide had evidence of significantly increased expansion of vaccine-reactive T cells as well as increased OS when compared with the non-cyclophosphamide cohort. There are also peptide-based vaccines under investigation, such as the multi-epitope anti-folate receptor vaccine that was used in combination with a PD-L1 inhibitor, Durvalumab, in a heavily pre-treated population; it showed promising results with one PR and nine SD [50]. Overall, these findings suggest the potential benefit of vaccines, at least as an adjunctive therapy in combination with other standard-of-care treatments.

## 4. Future Directions and Challenges

The ideal goal of ACT is to create a personalised and optimised cellular product with reactivity limited to the tumour. Over recent years, the manufacture of CAR-T, TCR-T and TILs has undergone multiple generations of development to improve their safety and efficacy as anti-cancer therapies. A challenge will be extrapolation of these approaches in OC, particularly as OC is generally recognised as having poor immunogenicity owing to a relatively low neoantigen burden [51]. The advantages are that many patients undergo major debulking surgery, which will hopefully allow for ample supplies of TILs. However, patients in more advanced settings of OC may not be fit enough for surgical resection of tissue. Less invasive strategies to obtain tumour-reactive T-cells, for example by leukapheresis or isolation from ascitic fluid, thus provide an alternative strategy in this cohort of patients and require further investigation [52,53,54,55].

The central challenge posed by engineered T-cell therapy is identifying suitable tumour-associated antigens with a low expression level in healthy tissue to maximise efficacy and reduce the burden of ‘on-target, off-tumour’ toxicity. Furthermore, as highlighted by the results of clinical studies, strategies are needed to enhance the survival of these cells *in vivo* and improve trafficking to the tumour site.

In the case of CAR-T therapy, there is evidence of a correlation between tumour burden and toxicity [56], so that attempts to reduce the tumour burden prior to ACT or dividing of doses may serve to reduce the risk of adverse effects. Additionally, newer generations of CAR-T cells have been modified to include ‘suicide genes’ or switches such as inducible caspase 9 [57], which has been incorporated into MUC1-C-targeted CAR-T cells in an ongoing study of patients with advanced epithelial derived cancers (NCT05239143; Table 2) [58]. There is evidence of an upregulation of inhibitory signals such as PD-1 in exhausted CAR-T cells, with a corresponding increase in inhibitory signals like PD-L1 on tumour cells [59,60]. Combinatorial approaches of CAR-T cell therapy with PD-1/PD-L1 ICIs have resulted in enhanced CAR-T cell activity, with 39% of patients having detectable CAR-T for >100 days peripherally and a 1-year overall survival of 83% in a phase 1 study looking at mesothelin CAR-T in malignant pleural disease [61]. Furthermore, methods to increase CAR-T efficacy by co-administration of oncolytic viruses and bispecific T-cell engagers are currently being evaluated [62]. Tumour-specific mutations, such as neoantigens [63], are fully foreign to the immune system, and are ideal targets for TCR-T therapies. Evaluation of these neoantigen-targeting TCR-T products are currently ongoing, with a successful case report in pancreatic cancer [64].

Finally, TILs are usually harvested as a heterogeneous group of cells and require further refinement to select for the most tumour-reactive lymphocytes, which are associated with enhanced anti-tumour reactivity [65]. Methods to enrich for antigen-reactive TILs through 4-1BB or PD-1 selection are currently being investigated in an effort to increase the efficacy of the final cell product delivered to patients [66,67]. Additionally, ‘young’ TILs have been associated with greater persistence *in vivo* and products that have undergone enrichment or a selection step for any of these tumour reactive populations are currently being evaluated in ongoing investigations [68,69,70]. Adjuvant IL-2 is typically administered following TIL therapy, but more novel systemic therapies, including ICIs, are increasingly being investigated as part of a combinatorial approach.

In the advanced and refractory setting, TIL harvesting may pose a significant challenge, given the paucity of TIL infiltration and/or the abundance of exhausted T-cells [71]; likely related to an immunosuppressive TME in these progressing tumours. This suggests that early harvest and delivery of a patient’s TILs may be more beneficial in their treatment journey [45]. Due to neoantigen editing and clonal evolution, manufacturing a cell product early with a delivery at a later time point could be problematic. However, this may be overcome by enriching for neoantigen-reactive T cells compared to delivering an unenhanced polyclonal TIL product [72].

## 5. Conclusions

Taken together, adoptive cellular therapies represent a personalised immunotherapeutic approach that has gained huge traction and development in recent years. The successes in the use of TIL therapy in melanoma [44] and CAR-T therapy in haematologic malignancies [15,16,17] have paved the way for ACT in solid tumours.

Unlike the widely used immune checkpoint inhibitors, ACT offers a more tumour-specific approach and can be tailored to the individual based on their tumour phenotype. Early clinical studies, although small, have been able to demonstrate evidence of disease control for ovarian cancer patients receiving ACT in the advanced setting. The use of combinations of these products with ICIs has shown yet more promising results with improved durable responses.

A disadvantage of engineered T-cell strategies over TIL is the need to further characterise suitable antigens to minimise ‘off-tumour’ side effects and increase ‘on-target’ efficacy. However, in the case of neuroblastomas, in which CAR-expressing T-cells targeted the disialoganglioside GD2, which are highly expressed on tumour cells, the right target was found, as the trial showed a phenomenal overall response of 63% with a 3-year OS of 60% in a refractory population with minimal off target toxicity [73]. Conversely, TIL therapy is advantageously ubiquitous if an ample supply of tumour sample is available, which may be a challenge in patients with advanced disease. Less invasive strategies could be explored to obtain tumour-reactive T-cells in this group of patients.

Beyond the clinical and scientific successes, ACT products are currently expensive, with increased regulatory oversight, as well as technically challenging to produce and deliver; this may limit future implementation on an international scale to reach ovarian cancer patients globally.

Although in the early stages of evaluation for solid malignancies, the evidence for ACT as a treatment option is encouraging, both alone and in combination with more well-established oncological therapies, for the improvement of outcomes for patients. Further optimization of this promising and exciting treatment modality is warranted to improve the anti-tumour effect and reduce the associated toxicity.

## Figures and Tables

**Table 1:** Published clinical trials of adoptive cellular therapy (ACT) in ovarian cancer (OC).

Target	Phase	Size	Preconditioning	Observations
CAR-T Mesothelin [23]	Phase I	n = 15 (OVCA, n = 5)	Cyclophosphamide	No CRS was observed 1 incident of grade 4 AE–sepsis Best overall response was SD (n = 11/15; including all 5 OVCA patients)
CAR-T Mesothelin [24]	Phase I	n = 15 (OVCA, n = 1)	None	No CRS or autoimmune toxicity was observed in any of the patients Peak blood levels at 7–14 days and evidence of CAR-T cells in metastases at 2 weeks but not 3–4 weeks 7/15 achieved SD 3–4 weeks post infusion
CAR-T Mesothelin	Phase I/II	n = 15	Cyclophosphamide/Fludarabine	Terminated due to slow accrual 14/15 patients had PD Result published on clinicaltrials.gov (NCT01583686)
CAR-T MUC16ecto [25]	Phase I	n = 18	Cyclophosphamide or cyclophosphamide with fludarabine	Some degree of CRS was observed at all doses DLT observed in 2/3 of the patients who received LD Best response was SD
CAR-T FRα [26]	Phase I	n = 14	None	Grade 3–4 toxicity in 5/8 patients who received IL-2 Grade 1–2 toxicity in group who received CAR-T cells without IL-2 T cells barely detectable in peripheral blood at 4 weeks in any patient No reduction in tumour burden in any patient
TCR MAGE-A4 [27]	Phase I	n = 38 (OVCA, n = 9)	Cyclophosphamide/Fludarabine	All patients experienced grade 3–4 haematological toxicity 55% patients had CRS across all tumour types 1 patient with OVCA had treatment-related death due to CVA after grade 3 neurotoxicity ORR was 24% 74% patients had SD or PR including 5 with OVCA Median duration of response was 25.6 weeks
TCR NY-ESO-1 [28]	Phase I	Estimated n = 22	Cyclophosphamide/Fludarabine	No DLT observed 2 PR, 5 SD and 1 PD at 2 years
TCR NY-ESO-1 [29]	Phase I/IIb	n = 6	Cyclophosphamide/Fludarabine	5/6 participants had serious AE 100% all-cause mortality at 12 months
TIL [30]	Phase I	n = 17	Cisplatin-based	1/7 CR and 4/7 PR in the TIL group 7/10 CR and 2/10 PR in the cisplatin-based chemotherapy + TIL group
TIL [31]	Phase I	n = 24	None	100% DFS at 3 years in TIL group compared with 67.5% in control group
TIL [32]	Phase I	n = 6	Cyclophosphamide	6/6 patients had SD at 6 weeks High levels of LAG-3, PD-1 were identified in infused T-cells
TIL [33]	Phase I/II	n = 6	Cyclophosphamide/Fludarabine	1/6 PR at 12 months 5/6 SD at 12 months

CAR-T, chimeric antigen receptor T-cell; TCR, T-cell receptor T-cell; TIL, tumour infiltrating lymphocyte; FRa, folate receptor alpha; MAGE-A4, melanoma associated antigen-A4; NY-ESO-1, New York esophageal antigen-1; OVCA, ovarian cancer; CRS, cytokine release syndrome; AE, adverse effects; SD, stable disease; DLT, dose limiting toxicity; LD, lymphodepletion; CVA, cerebrovascular accident; ORR, overall response rate; PR, partial response; PD, progressive disease; CR, complete response; DFS, disease free survival; LAG-3, lymphocyte activation gene-3; PD-1, programmed death-1.

**Table 2:** Selected ongoing clinical trials of adoptive cellular therapy (ACT) in ovarian cancer (OC).

Target	Phase	Size	Preconditioning	Status (Estimated Primary Completion Date)	Clinical Trial Number
CAR-T Mesothelin	Phase I	n = 10	Cyclophosphamide/Fludarabine	Unknown (January 2023)	NCT03814447
CAR-T Mesothelin	Phase I/II	n = 3	None	Closed for accrual (February 2023)	NCT05372692
CAR-T Mesothelin	Phase I/II	Estimated n = 20	Cyclophosphamide/Fludarabine	Unknown (April 2023)	NCT03916679
CAR-T Mesothelin	Phase I/II	Estimated n = 10	Cyclophosphamide/Fludarabine	Open for accrual (May 2026)	NCT05963100
CAR-T MUC16	Phase I	Estimated n = 71	Cyclophosphamide	Open for accrual (November 2028)	NCT03907527
CAR-T MUC1-C	Phase 1	Estimated n = 100	Unknown	Open for accrual (April 2026)	NCT05239143
CAR-T MOV19-BBZ	Phase I	Estimated n = 12–24	Cyclophosphamide/Fludarabine	Open for accrual (October 2028)	NCT03585764
CAR-T TAG-72	Phase I	Estimated n = 33	Cyclophosphamide/Fludarabine	Open for accrual (April 2027)	NCT05225363
CAR-T B7H3	Phase I	Estimated n = 25	Cyclophosphamide/Fludarabine	Open for accrual (February 2025)	NCT04670068
CAR-T B7H3	Phase I/II	Estimated n = 15	Cyclophosphamide/Fludarabine	Open for accrual (August 2026)	NCT05211557
CAR-T ALPP	Phase I/II	Estimated n = 20	Cyclophosphamide/Fludarabine	Closed for accrual (December 2022)	NCT04627740
TCR MAGE-A4	Phase II	Estimated n = 66	Cyclophosphamide/Fludarabine	Open for accrual (October 2025)	NCT05601752
TCR NY-ESO-1-	Phase I	n = 9	Cyclophosphamide/Decitabine	Closed for accrual (March 2020)	NCT03017131
TCR NY-ESO-1	Phase I	n = 4	Melphalan	Closed for accrual (January 2021)	NCT03691376
TCR Neoantigens	Phase II	Estimated n = 270	Cyclophosphamide/Fludarabine	Open for accrual (March 2027)	NCT03412877
TIL	Phase II	Estimated n = 15	Fludarabine Radiotherapy	Unknown (February 2021)	NCT03412526
TIL	Phase I	n = 3	Cyclophosphamide	Closed for accrual (June 2023)	NCT01883297
TIL in combination with nivolumab, relatlimab and ipilimumab	Phase I/II	Estimated n = 18	Cyclophosphamide/Fludarabine	Open for accrual (December 2022)	NCT04611126
TIL [34] in combination with pembrolizumab	Phase I	n = 8	Cyclophosphamide/Fludarabine	Closed for accrual (August 2020)	NCT03158935
TIL in combination with IFNα	Phase I/II	Estimated n = 12	Carboplatin/Paclitaxel	Unknown (August 2021)	NCT04072263

CAR-T, chimeric antigen receptor T-cell; TAG-72, tumour-associated glycoprotein-72; MAGE-A4, melanoma-associated antigen-A4; NY-ESO-1, New York esophageal antigen-1; TCR, T-cell receptor T-cell; TIL, tumour-infiltrating lymphocyte; IFNα, interferon alpha.

## Data Availability

The data presented in this study are available in this article.
